# Global research trends of steroid-induced osteonecrosis of the femoral head: A 30-year bibliometric analysis

**DOI:** 10.3389/fendo.2022.1027603

**Published:** 2022-10-17

**Authors:** Chao Lu, Haodong Qi, Hanbo Xu, Yangquan Hao, Zhi Yang, Wenxing Yu, Peng Xu

**Affiliations:** ^1^ Department of Joint Surgery, Honghui Hospital, Xi’an Jiaotong University, Xi’an, China; ^2^ Graduate School, Shaanxi University of traditional Chinese Medicine, Xi’an, China

**Keywords:** steroid-induced osteonecrosis of the femoral head, SONFH, bibliometric analysis, trends, hotspots

## Abstract

**Objective:**

To explore the global research trends and hotspots of steroid-induced osteonecrosis of the femoral head (SONFH) through qualitative and quantitative analysis of bibliometrics.

**Methods:**

All publications on SONFH published from 1992 to 2021 were extracted from the Web of Science Core Collection database. CiteSpace was used for the visualization analysis of major countries, active institutions, productive authors, and the burst of keywords. VOSviewer was used for coupling analysis of countries/regions, institutions, and authors. Microsoft Excel 2017 was used for statistical analysis, drawing bar charts, pie charts, and cumulative area charts. The software of MapInfo was used to draw the distribution map of the publications.

**Results:**

A total of 780 publications were included for analyses. The most productive year was 2020 with 98 records. China was the most influential country with 494 publications, an H-index of 59, and total citations of 16820. The most prolific institution was Shanghai Jiaotong University in China with 53 publications and 998 citations. *Clinical Orthopaedics and Related Research* (IF = 4.755, 2021) was the most active journal with 26 articles. The hot keywords were “osteonecrosis”, “avascular necrosis”, “osteogenic differentiation”, “proliferation”, “PPAR gamma”, “apoptosis”, “oxidative stress”, “genetic polymorphism” and “mesenchymal stem cells”. The keywords like “proliferation”, “PPAR gamma” and “genome-wide” have emerged in recent years.

**Conclusion:**

The number of publications in SONFH has increased significantly in the last three decades. The pathologic mechanism of SONFH gathered most research interests. Genomics and cell molecular biology of SONFH are the research frontiers.

## Introduction

Osteonecrosis of the femoral head (ONFH) is a widespread disabling pathology that mostly affects the young and middle-aged population and is one of the major causes of total hip arthroplasty in the elderly (1). ONFH can be clinically divided into traumatic and nontraumatic types based on diverse etiologies. Steroids can cause the death of bone dynamics components and are the most common cause of ONFH (2, 3). Long-term high-dose use of glucocorticoids is the key risk factor for nontraumatic femoral head necrosis (4, 5), which was known as steroid-induced osteonecrosis of the femoral head (SONFH). With the widespread use of glucocorticoids in the treatment of rheumatic immune diseases (6, 7), SONFH was commonly seen in clinical, with the incidence up to 40% (8, 9). SONFH is bilateral and symmetrical, with a wide range of necrosis and a high disability rate, which brings a catastrophic burden to patients and their families. Although there are several theories about the pathogenesis of SONFH, such as apoptosis of bone and osteoblasts (10, 11), osteolytic bone destruction (12), coagulation disorder and intravascular thrombosis (13), endothelial cell apoptosis and angiogenesis disorders (14–16), bone marrow fat accumulation and intramedullary pressure change (17), which eventually lead to the death of cells in the femoral head due to ischemia and hypoxia, and then caused the subchondral collapse and necrosis of femoral head, the exact mechanism is still unclear. Driven by these controversies, SONFH has piqued the interest of global researchers, and the number of related publications has increased rapidly in recent decades. To date, however, only a few bibliometric analyses have investigated this area. Further systematic research on the knowledge structure, research trends, and hotspots of SONFH will be helpful for the researchers to understand this field comprehensively.

Bibliometrics is an interdisciplinary science integrating mathematical and statistical methods. It can conduct qualitative and quantitative analyses of publications in a certain research domain, to analyze research status and research hotspots. In addition, it can predict the research frontier and future development trends based on the literature and knowledge field characteristics. This study aims to summarize the current research status of SONFH, analyze its cooperation network, and identify the main research trends and hotspots.

## Methods

### Retrieval strategies and data collection

**Figure 1:**
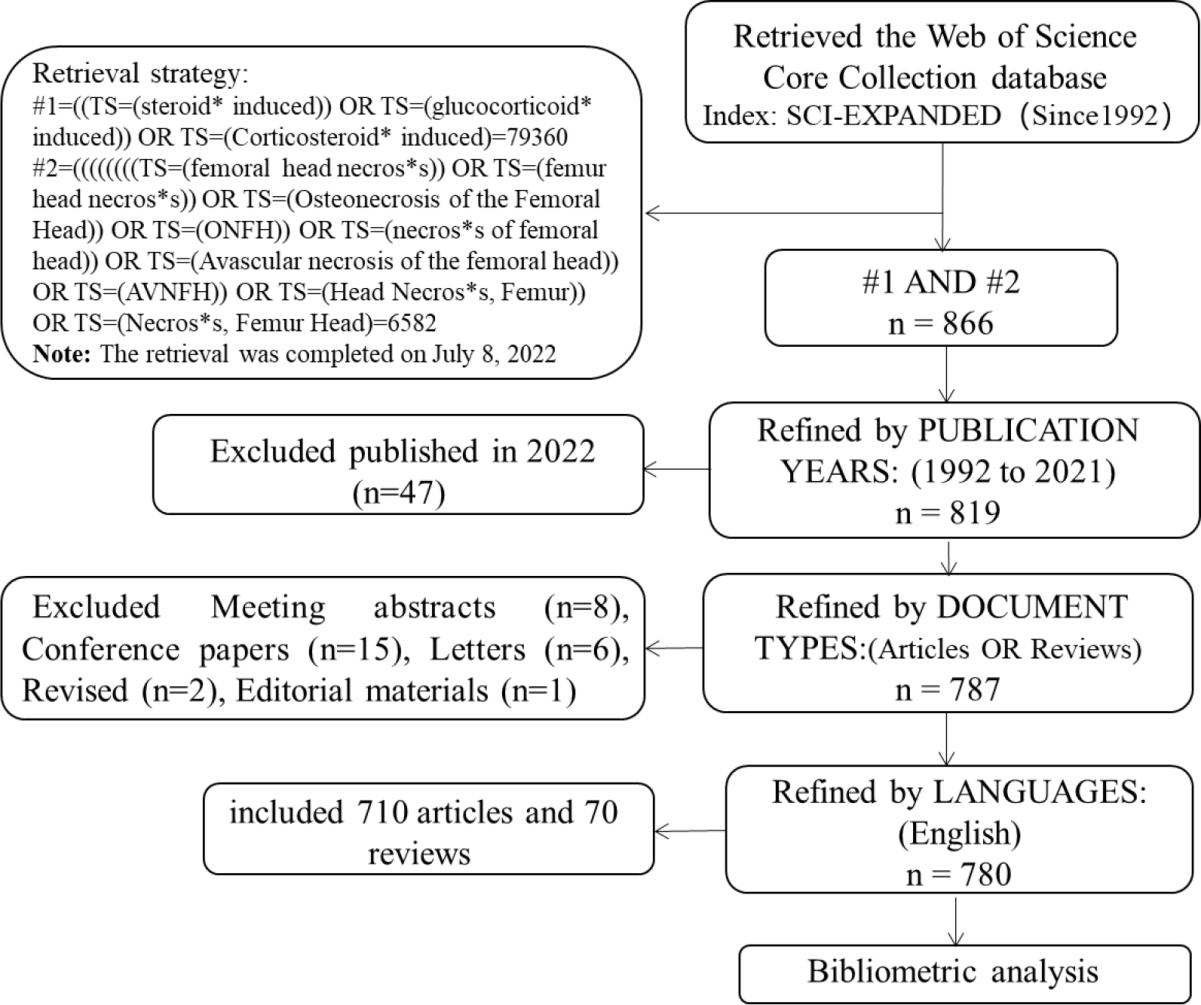
Flow chart of SONFH-related literature screening.

As the main data source, we selected the Science Citation Index Expanded (SCI-Expanded) of the Web of Science Core Collection (WoSCC). Relevant literature on SONFH was retrieved on July 8, 2022. Before the retrieval, medical subject headings (MeSH) and their entry words in PubMed developed by the National Biotechnology Information Center (NCBI) of the National Library of Medicine (NLM) were combined to comprehensively and accurately search the topic. The retrieval strategies were as the following: TS (TOPICS) =((steroid* induced) OR (glucocorticoid* induced) OR (Corticosteroid*induced)) AND TS= ((femoral head necros*s) OR (femur head necros*s) OR (Osteonecrosis of the Femoral Head) OR (ONFH) OR (necros*s of femoral head) OR (Avascular necrosis of the femoral head) OR (AVNFH) OR (Head Necros*s, Femur) OR (Necros*s, Femur Head)), the timespan was set from 1992 to 2021. The final 780 articles were included by Refined: [DOCUMENT TYPES: (Articles OR Reviews) AND LANGUAGE: (English)]. The literature retrieval process was shown in **Figure 1**.

The full records and references cited included in the study were exported from the WoSCC database in plain text format, including titles, years of publications, authors, countries, author’s institutions, journals of publications, abstracts, keywords, the total number of publications, sum times of cited, average citations per item and H-index. The exported file was named “download.txt”. The “download.txt” file was imported into bibliometrics software for qualitative and quantitative analysis. The journal impact factors (IF) and quartile ranks were collected from the 2021 Journal Citation Reports.

### Bibliometric analysis

Two bibliometric tools were used to comprehensively analyze SONFH-related publications: VOSviewer (1.6.18 edition, https://www.vosviewer.com/) and CiteSpace (6.1.R2 Basic edition, http://cluster.cis.drexel.edu/~cchen/citespace/download/). VOSviewer software was used to conduct bibliographic coupling analysis of countries, institutions, and authors, and co-occurrence analysis of keywords. The size of the nodes indicated the number of publications, the thickness of the line represented the strength of the relationship, and the colors of the nodes showed distinct clusters or periods. CiteSpace was used to carry out cluster analysis and citation burst of keywords, which was helpful to detect the research hotspots and development trends. Microsoft Excel 2017 was used for statistical analysis, drawing bar charts, pie charts, and cumulative area charts. The software of MapInfo was used to draw the distribution map of the publications.

## Results

### Publication outputs and trends

**Figure 2:**
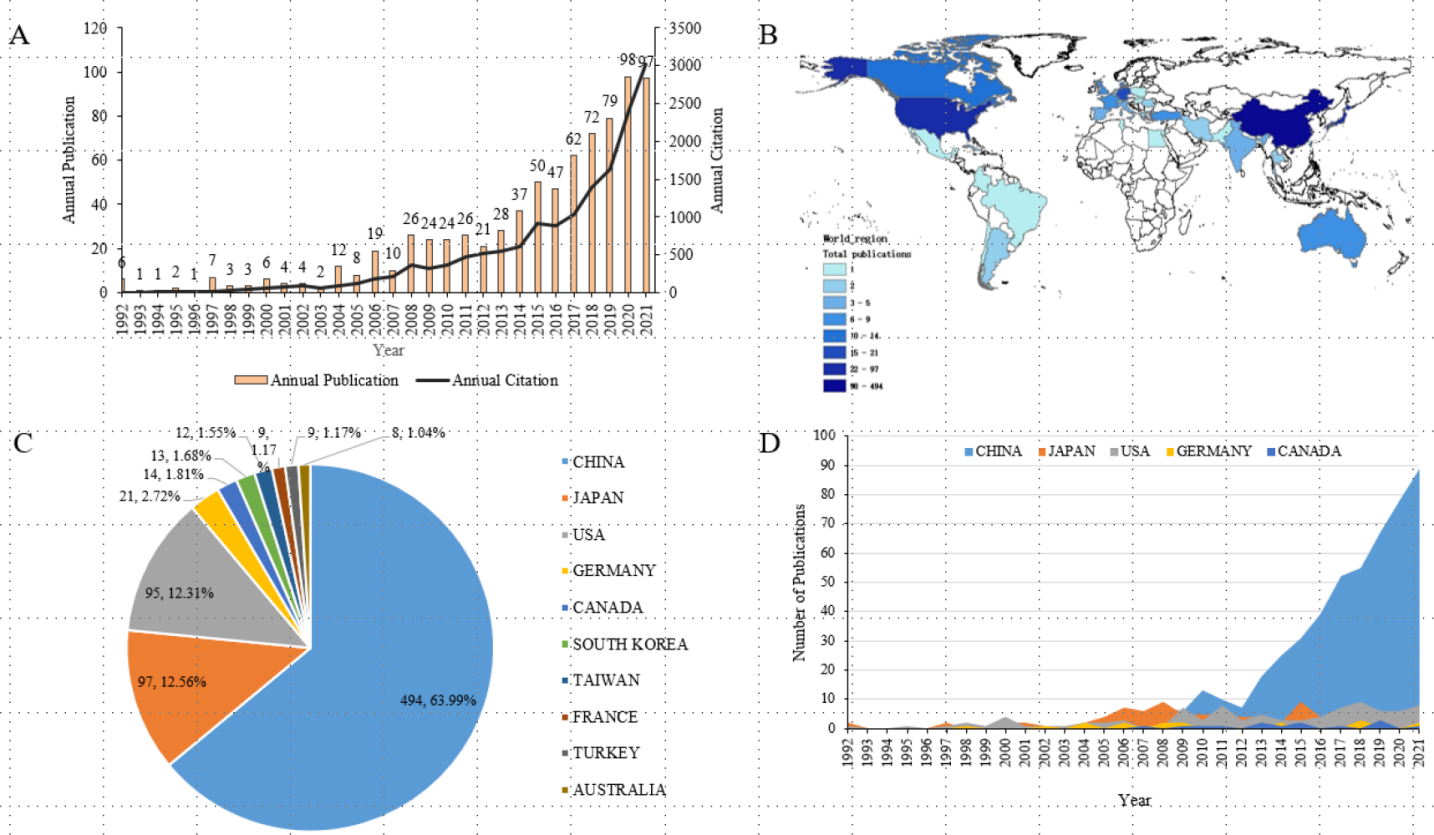
Global trends of publications on SONFH in the last 30 years. **(A)** Annual publications and annual citations on SONFH from 1992 to 2021. **(B)** Geographical distribution of publications on SONFH from 1992 to 2021. **(C)** The total number and percentage of publications from the top 10 countries/regions. **(D)** The annual publications in the top 5 prolific countries/regions from 1992 to 2021.

**Figure 2** listed the outputs and global trends of publications on SONFH in the last 30 years. A total of 780 papers relevant to SONFH were included in our study and the annual publications have grown nearly 16-fold over the past three decades from 6 in 1992 to 97 in 2021 (**Figure 2A**). The most productive year was 2020 with 98 records and the most cited year was 2021 with 3022 cited times. The annual publications did not exceed 10 until 2004, and then gradually increased with fluctuations from 2004 to 2012. In the last decade, except for 2021, the annual publications had shown a steady and rapid growth from 21 in 2012 to 98 in 2020. The total citations of the 780 papers were 16817, and 11592 after excluding self-citations. The average citations were 21.56 and the h-index was 59. The trend of annual citations was consistent with that of annual publications. A total of 37 countries/regions published papers on SONFH, and **Figure 1B** showed the heatmap distribution by the number of publications in each country/region. The top 10 countries/regions by the number of publications were shown in **Figure 2C**. China ranked first in the number of total publications (494, 63.99%), followed by Japan (97, 12.56%), the USA (95, 12.31%), Germany (21, 2.72%), and Canada (14, 1.81%). China produced five times literature more than Japan and the USA, indicating that China was a high-yield country and paid more attention to SONFH research. **Figure 2D** showed the annual publication trends of the top 5 countries in the last 30 years. In the past decade, China’s annual publications showed a rapid straight upward trend.

### Analysis of the research areas and productive journals

Journal Citation Reports (JCR) is an effective tool for systematic and objective evaluation of the world’s authoritative journals. The division of JCR journals is based on journal impact factors (JIF) included in SCI (Science Citation Index) and SSCI (Social Science Citation Index). JCR profiles consist of four indicators, including impact factor, category, ranking, and quartile (Q), representing a journal’s position and influence in academic communication networks.

**Figure 3:**
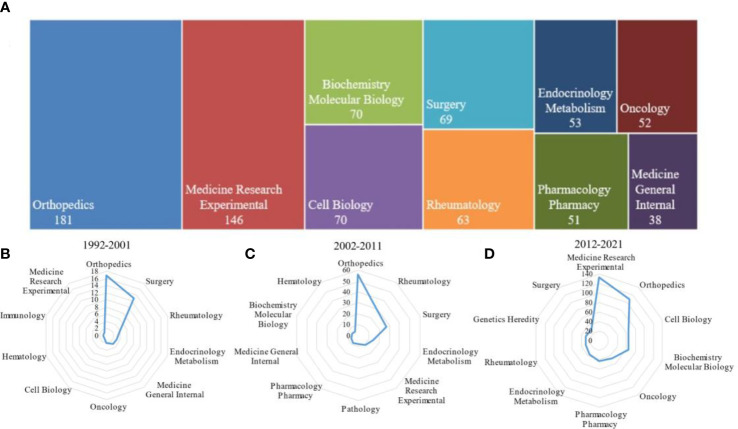
Research area analyses of global publications in SONFH in the past three decades. **(A)** The top 10 research areas on SONFH from 1992 to 2021. **(B)** Radar map of the top 10 research productive categories in SONFH during 1992-2001. **(C)** Radar map of the top 10 research productive categories in SONFH during 2002-2011. **(D)** Radar map of the top 10 research productive categories in SONFH during 2012-2021.

The subject category of Web of Science (WOS) was divided into 254 subject areas according to journals. In our study, 780 papers related to SONFH from 1992 to 2021 were published in 312 journals and categorized into 75 different research areas on WOS (**Supplementary Material 1**). Nine research areas contained more than 50 publications. The top 10research areas and the corresponding number of SONFH-related publications were presented in **Figure 3A**. The orthopedics area got the most attention (181 records, 23.205%), followed by medicine research experimental (146 records, 18.718%), biochemistry molecular biology (70 records, 8.974%), cell biology (70 records, 8.974%), surgery (69 records, 8.846%), rheumatology (63 records, 8.077), endocrinology Metabolism (53 records, 6.795), oncology (52 records, 6.667), pharmacology Pharmacy (51 records, 6.538) and medicine general internal (38 records, 4.872). **Figure 3B, C, and D** displayed the top 10research areas during 1992-2001, 2002-2011, and 2012-2021, respectively. It can be seen from the development and change trend of discipline categories in SONFH that “medicine research experimental” had attracted more and more attention in the past 30 years, rising from 10th place during 1992-2001 to fifth place during 2002-2011 and reaching the first place during 2012-2021. Biochemistry molecular mechanisms related to SONFH were also thoroughly explored, and research interest in the “biochemistry molecular biology” and “cell biology” areas has continued to increase over the past 20 decades. On the contrary, it was observed that the popularity of the research areas of “surgery”, “rheumatology” and “endocrinology metabolism” related to SONFH was decreasing.

The top 10 most productive journals were listed by the number of publications in **Table 1**. Among the top 10 productive journals, four publishers of these journals were from the United States, four from the UK, and two from Greece. The top 10 journals were distributed into five main categories: Orthopedics, Medicine, Research & Experimental, Endocrinology& Metabolism, Biochemistry& Molecular Biophysics, and Rheumatology. In the journal category, five of the top 10 journals were assigned to orthopedics. *Clinical Orthopaedics and Related Research* (IF = 4.755, 2021) was the most active journal in SONFH research, with 26 articles, followed by *International Orthopaedics* (IF = 3.479, 2021), with 19 articles; *Molecular Medicine Reports* (IF = 3.423, 2021), with 19 articles; *BMC Musculoskeletal Disorders* (IF = 2.562, 2021), with 17 articles; and *Experimental and Therapeutic Medicine* (IF = 2.751, 2021), with 16 articles. *Clinical Orthopaedics and Related Research* ranked first in the publications of the journals (26 records), h-index ranking (18), and total citations (1167 times), and average citations (44.88 times). *Rheumatology* ranked 10th in the number of publications, while it had the highest impact factor (IF = 7.046, 2021). We can track the latest progress of SONFH by monitoring the latest studies in the top 10 journals.

**Table 1:** Top 10 productive journals on SONFH from 1992 to 2021.

Rank	Journal^a^	Number	Country	h-index	Total citations	Average citations	JCR			
							IF2021^b^	Category	Rank	Quartile
1	Clinical Orthopaedics and Related Research	26	United States	20	1167	44.88	4.755	OrthopedicsSurgery	11/8630/211	Q1Q1
2	International Orthopedics	19	United States	13	459	24.16	3.479	Orthopedics	25/86	Q2
3	Molecular Medicine Reports	19	Greece	10	221	11.63	3.423	Medicine,Research & ExperimentalOncology	87/139160/245	Q3Q3
4	BMC Musculoskeletal Disorders	17	UK	10	307	18.06	2.562	OrthopedicsRheumatology	44/8628/34	Q3Q4
5	Experimental and Therapeutic Medicine	16	Greece	6	121	7.56	2.751	Medicine, Research & Experimental	105/139	Q4
6	Journal of Orthopaedic Research	14	UK	10	410	29.29	3.102	Orthopedics	31/86	Q2
7	Bone	13	United States	9	427	32.85	4.626	Endocrinology& Metabolism	60/146	Q2
8	Biochemical and Biophysical Research Communications	13	United States	8	284	21.85	3.322	Biochemistry& MolecularBiophysics	196/29639/72	Q3Q3
9	Journal of Orthopaedic Surgery and Research	13	UK	5	69	5.31	2.677	Orthopedics	43/86	Q2
10	Rheumatology	12	UK	10	418	34.84	7.046	Rheumatology	6/34	Q1

a journal names according to the Index of Medical Journal Abbreviations;

b IF in the category according to Journal Citation Reports (2021).

IF, impact factor.

### Analysis of highly cited articles

The papers with high citations can objectively reflect that the research has raised attention from the scholars, and to some extent, also represent the author’s academic influence. **Table 2** listed the top 10 highest cited articles in the past 30 years in descending order by the total number of citations. The top 10 most cited articles with 7 research articles and 3 reviews were published from 1997 to 2020 and the number of total citations ranged from 171 to 301. The article ranked first was Weinstein’s study on the pathogenesis of SONFH, published in “*J Clin Endocrinol Metab*” in 2000 (IF=6.134, Total citations=301, Average citations=13.09). This article suggested that glucocorticoid-induced osteocyte apoptosis was the main mechanism of hip joint lesions. The article with the highest average citations was published in “*Bone Res*” in 2020 (IF=13.362, Total citations=198, Average citations=66). This was a 15-year follow-up study of SARS (severe acute respiratory syndrome) patients treated with high-dose steroid pulse therapy, which showed that SONFH in SARS patients was not progressive and was partially reversible. From another perspective, the highest citation frequency of this article, published only 2 years ago, indicates that researchers have been paying continuous attention to the long-term outcome of femur head necrosis caused by a high dose of steroids. Among the top highest cited articles, the article published in *“Blood”* in 2011 had the highest impact factor (IF=25.476, Total citations=177, Average citations=14.75), it was a study on pharmacokinetics, pharmacodynamics, and pharmacogenetic determinants of osteonecrosis in children with acute lymphoblastic leukemia, and the results showed that older age, lower albumin levels, higher lipid levels, and dexamethasone exposure were associated with osteonecrosis and may be associated with the inherited genomic variation.

**Table 2:** Top 10 highest cited articles on SONFH from 1992 to 2021.

Rank	First author	Article	Study type	IF (2021)	Total citations	Average citations
1	Weinstein, RS	Apoptosis of osteocytes in glucocorticoid-induced osteonecrosis of the hip. *J Clin Endocrinol Metab*, 2000, 85(8):2907-12.	Article	6.134	301	13.09
2	Mont, MA	Nontraumatic Osteonecrosis of the Femoral Head: Where Do We Stand Today? *J Bone Joint Surg Am*, 2015, 97(19):1604-27.	Review	6.558	253	31.63
3	Kerachian, MA	Glucocorticoids in osteonecrosis of the femoral head: A new understanding of the mechanisms of action. *J Steroid Biochem Mol Biol*, 2009, 114(3-5):121-8.	Review	5.011	231	16.5
4	Weinstein, RS	Glucocorticoid-Induced Osteoporosis and Osteonecrosis. *Endocrinol Metab Clin North Am*, 2012, 41(3):595-611.	Article	4.748	225	20.45
5	Weinstein, RS	Glucocorticoid-induced osteonecrosis. *Endocrine*, 2012, 41(2):183-90.	Review	3.925	213	19.36
6	Zhang, PX	Long-term bone and lung consequences associated with hospital-acquired severe acute respiratory syndrome: a 15-year follow-up from a prospective cohort study. *Bone Res*, 2020, 14; 8:8.	Article	13.362	198	66
7	Yamamoto, T	Effects of pulse methylprednisolone on bone and marrow tissues - Corticosteroid-induced osteonecrosis in rabbits. *Arthritis Rheum*, 1997, 40(11):2055-64.	Article	8.955	189	7.27
8	Kawedia, JD	Pharmacokinetic, pharmacodynamic, and pharmacogenetic determinants of osteonecrosis in children with acute lymphoblastic leukemia. *Blood*, 2011, 117(8):2340-7.	Article	25.476	177	14.75
9	Pritchett, JW	Statin therapy decreases the risk of osteonecrosis in patients receiving steroids. *Clin Orthop Relat Res*. 2001, (386):173-8.	Article	4.755	176	8
10	Hernigou, P	Decrease in the mesenchymal stem-cell pool in the proximal femur in corticosteroid-induced osteonecrosis. J *Bone Joint Surg Br*, 1999, 81(2):349-55.	Article	3.309	171	7.13

### Analysis of the influential countries/regions, institutions, and authors

**Figure 4:**
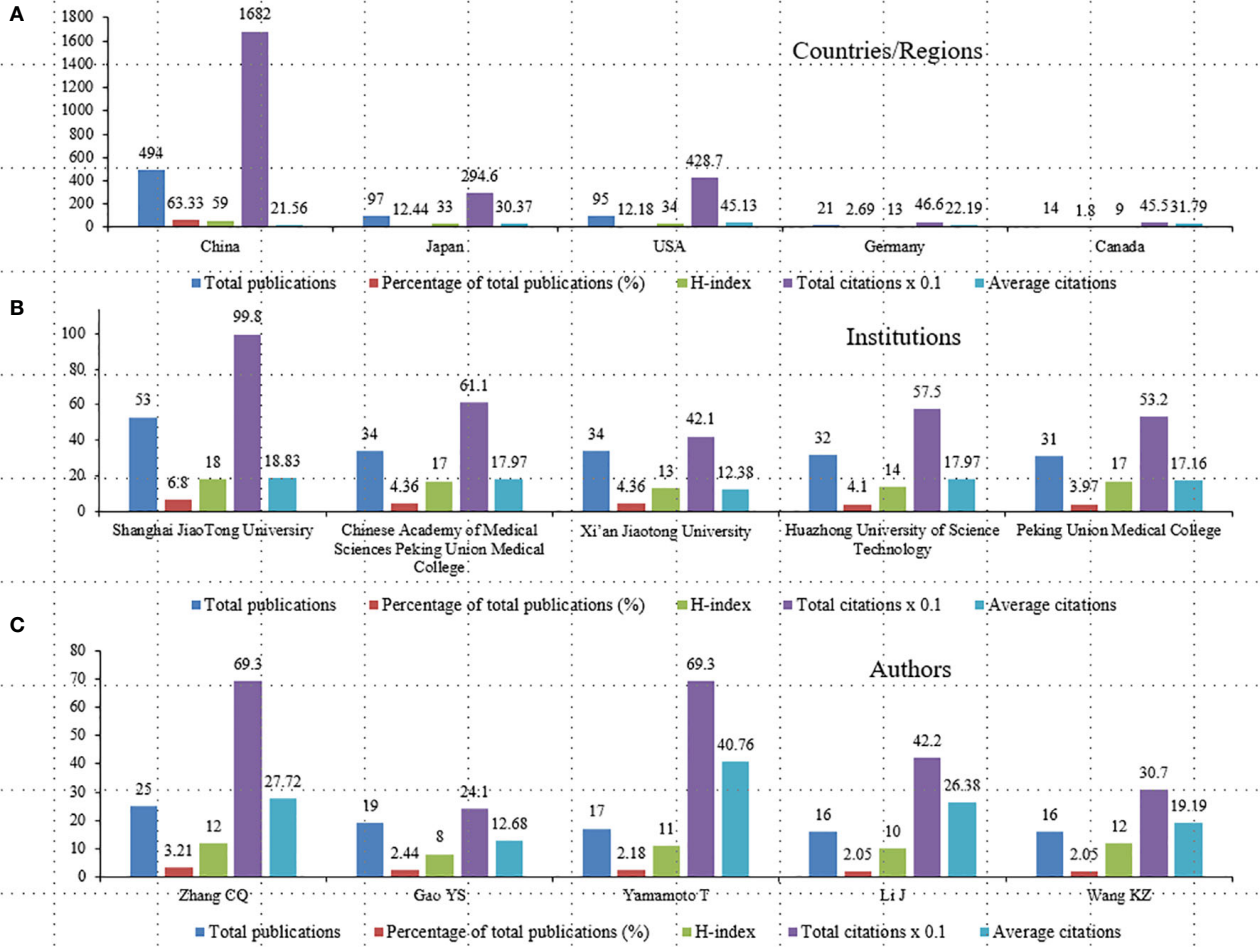
The Top 5 productive countries/regions, institutions, and authors on SONFH from 1992 to 2021. **(A)** Total publications, percentage of total publications (%), H-index, total citations, and average citations per item of the top 5 countries/regions by contributions; **(B)** Total publications, percentage of total publications (%), H-index, total citations, and average citations per item of the top 5 institutions by contributions; **(C)** Total publications, percentage of total publications (%), H-index, total citations, and average citations per item of the top 5 authors by contributions.

Analysis of the most productive and influential countries, institutions, and authors can help academics understand existing cooperative relationships and identify potential partners (19). **Figure 4** illustrated the total publications, percentage of total publications (%), h-index, total citations, and average citations per item of the top 5productive countries/regions, institutions, and authors with the most publications related to SONFH. The 780 articles in our study were distributed in 37 countries/regions, while the top 5countries/regions published 721 papers accounting for 92.44% (**Figure 4A**, **Supplementary Table 1**). China was the most prolific country with 494 publications, and the highest h-index of 59, with total citations of 16820. The United States achieved the highest average citations of 45.13 among the 37 countries/regions. Germany obtained the highest centrality of 0.24 (**Supplementary Table 1**), illustrating that Germany had played a good role as an academic bridge in this research field.

The top 5institutions published 184 papers accounting for 23.59%, of which all came from China. Shanghai Jiaotong University ranked first with 53 publications, followed by the Chinese Academy of Medical Sciences Peking Union Medical College (34 records), Xi’an Jiaotong University (34 records), Huazhong University of Science Technology (32 records), and Peking Union Medical College (31 records). Shanghai Jiaotong University and Peking Union Medical College both got the highest h-index of 17 (**Figure 4B**, **Supplementary Table 1**).

The top 5authors published 93 papers accounting for 11.92%. Zhang CQ, from the Department of Orthopedics Surgery, Shanghai Jiaotong University Affiliated Sixth Hospital, took the top position with 25 publications, and he and Wang KZ from the Department of Orthopedics Surgery, Xi’an Jiaotong University tied for first with h-index 12. Yamamoto T from Fukuoka University had the highest average citations of 40.76 among the 2933 authors, and he and Zhang CQ ranked co-first with 693 total citations (**Figure 4C**, **Supplementary Table 1**).

### Cooperative network analysis of the prolific countries/regions, institutions, and authors

Bibliographic coupling analysis is a method to show the correlation between items according to the number of references shared by the items. To some extent, the total link strength of a particular item can explain its global influence. An item with higher total link strength indicates more influential (18). This study used bibliographic coupling analysis to establish a similar relationship among publications from three dimensions: country, institution, and author.

**Figure 5:**
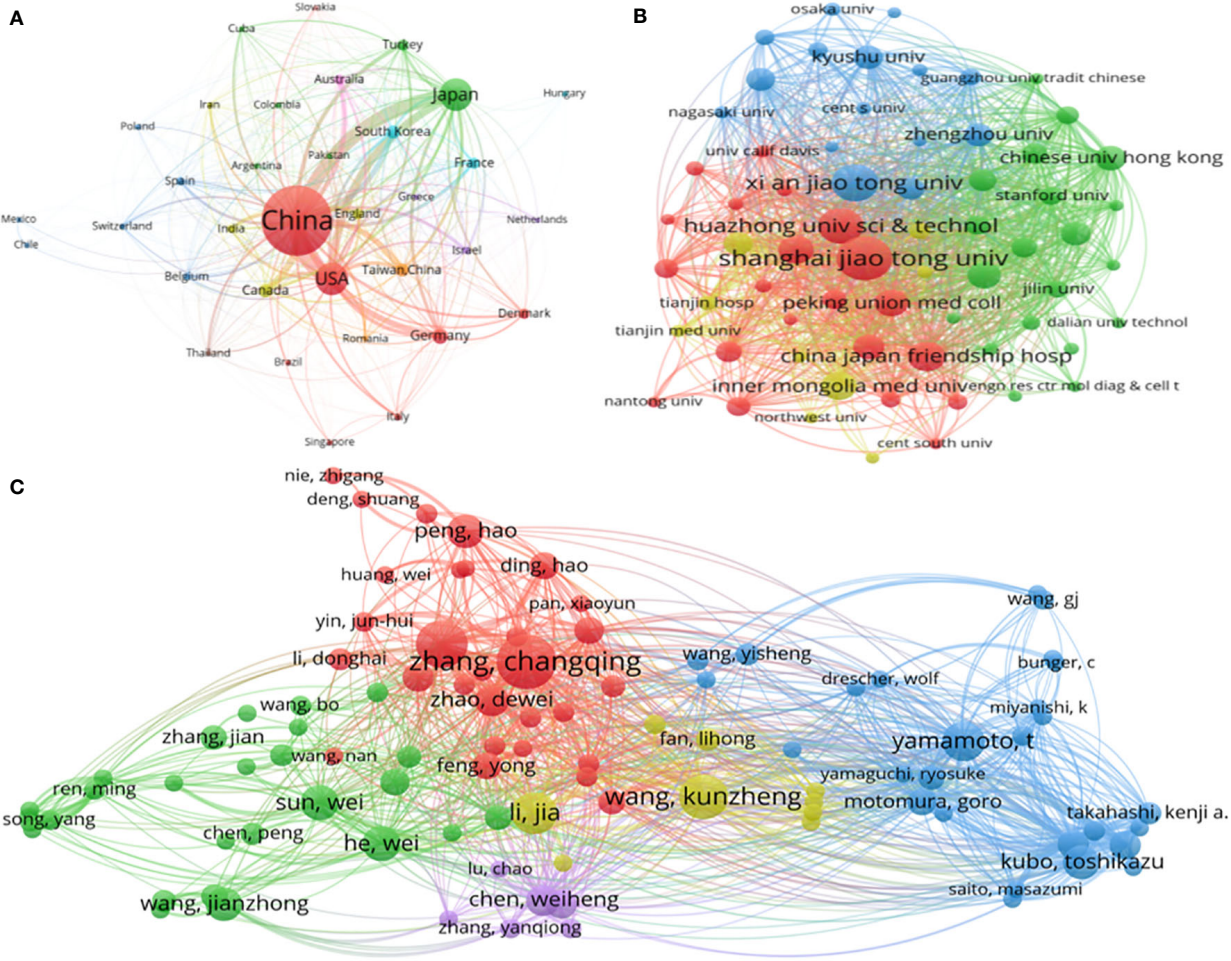
Bibliographic coupling analyses of global publications in SONFH in the last 30 years. **(A)** Network visualization of the 35 identified countries/regions in SONFH. **(B)** Network visualization of the 65 identified institutions in SONFH. **(C)** Network visualization of the 102 identified authors in SONFH. Each circle represents an item; the size of the circle represented the number of publications of the item. The distance between two circles represents the correlation of the items, a closer distance with a stronger association. The color of an item is determined by the cluster to which it belongs.

**Figure 5A** exhibited the cooperative relationships of 24 identified countries (the minimum number of documents of a country was over two) using the bibliographic coupling analysis of VOSviewer. In order of the total link strength (TLS), China ranked first with the TLS of 68495, 494 documents, and 7024 citations, followed by the United States (TLS = 40772, citations = 4121, documents = 95), Japan (TLS = 30354, citations = 2547, documents = 97), Canada (TLS = 7447, citations = 445, documents = 14) and Germany (TLS = 7272, citations = 415, documents = 21). According to the bibliographic coupling analysis, China was the most influential country in global SONFH research, consistent with the highest total number of publications, total citations, and H-index of China in SONFH described above.

The cooperation relationship of 65 identified institutions (the minimum number of documents of an institution was over five) was displayed in **Figure 5B** by TLS using VOSviewer. The top 5 institutions in terms of TLS were as follows: Shanghai Jiaotong University (TLS = 16538, citations = 939, documents = 53), Huazhong University of Science Technology (TLS = 12650, citations = 575, documents = 32), Xi’an Jiaotong University (TLS = 12334, citations = 421, documents = 34), China-Japan Friendship Hospital (TLS = 11535, citations = 417, documents = 24), and Peking Union Medical College (TLS = 10273, citations = 346, documents = 31). According to the bibliographic coupling analysis, Shanghai Jiaotong University was the most influential institution in global SONFH research, consistent with the highest total number of publications, total citations, average citations, and H-index of Shanghai Jiaotong University in SONFH discussed earlier.

**Figure 5C** represented the cooperation relationship of 102 identified authors (the minimum number of documents of an author was over five) in TLS using VOSviewer. The top 5authors in terms of TLS were as follows: Zhang CQ (TLS = 12119, citations = 639, documents =23), Yamamoto T (TLS = 9409, citations = 631, documents =15), Wang KZ (TLS = 9139, citations = 321, documents =18), Gao YS (TLS = 8453, citations = 241, documents =19), and Kubo T (TLS = 7706, citations = 259, documents =12). Therefore, Zhang CQ, Yamamoto T, and Wang KZ were the top three influential contributors to SONFH, which matched the results described earlier.

### Analysis of research hotspots and trends

**Figure 6:**
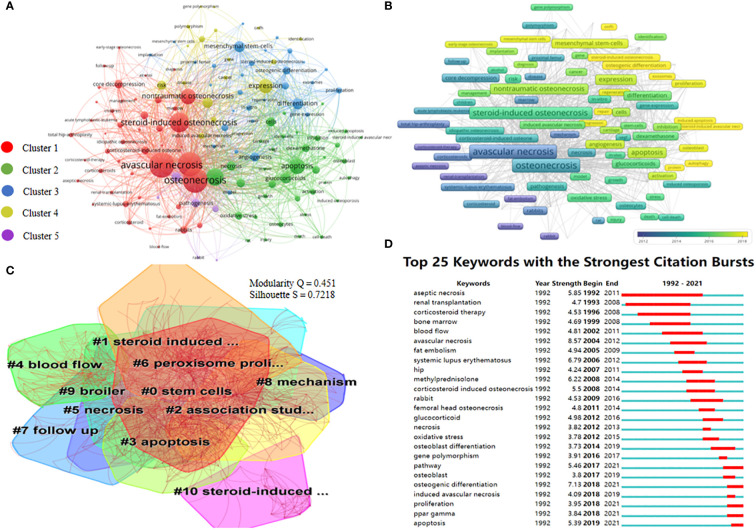
Keywords co-occurrence knowledge graph. **(A)** Keywords co-occurrence network visualization on SONFH from 1992 to 2021. Each node represents one keyword, the node size represented the frequency of a keyword, larger size with a higher frequency. The distance between nodes represents the correlation of two keywords, a closer distance with a stronger association. **(B)** Keywords co-occurrence overlay visualization on SONFH from 1992 to 2021. The node color represents the average publication year of a keyword. The colors vary from dark blue, turquoise, green, and green-yellow to yellow from far to near with the year. **(C)** Keywords cluster atlas by CiteSpace. The cluster labels were numbered from 0 to 17. The smaller the number, the more keywords it contained. **(D)** The top 25 keywords with the strongest citation bursts. The years between “Begin” and “End” indicate the duration of a keyword burst. The blue line indicates that the keyword has not yet appeared, and the red line indicates that the keyword has a strong influence.

Keywords are an important indicator of research topics and hotspots, and also a highly refined summary of research content (20). Keywords co-occurrence network can reflect the research hotspots in a certain field. The overlay visualization map of VOSviewer can divide keywords into different clusters based on co-occurrence analysis, and mark keywords with different colors according to the temporal evolution. A total of 2509 keywords were detected in our study, among which 135 terms that appeared more than 10 times were separated into five clusters (**Figure 6A**, links=4519, TLS=14927). The five clusters in different colors of red, green, blue, yellow, and purple represent cluster 1 (45 items), cluster 2 (36 items), cluster 3 (30 items), cluster 4 (18 items), and cluster 5 (6 items), respectively. The top 10 keywords with the highest co-occurrence frequency were osteonecrosis (253 times), avascular necrosis (250 times), femoral-head (238 times), steroid-induced osteonecrosis (179 times), bone (151 times), apoptosis (128 times), nontraumatic osteonecrosis (128 times), expression (113 times), differentiation (80 times) and mesenchymal stem cells (79 times). Through in-depth analysis of high-frequency keywords and their correlation strength, it can be inferred that ischemic femoral head necrosis, apoptosis mechanism, and mesenchymal stem cell therapy were the research hotspots of SONFH in the last 30 years.

The keywords co-occurrence overlay visualization map was displayed in **Figure 6B**. The color of the keywords depends on the average year they appear in the publications, the yellow keywords appear later, representing the latest research hotspots to some extent. In this study, the research focus in recent 10 years had gradually shifted from the study of the pathogenesis of ischemic necrosis to the study of stem cell treatment methods over time, such as osteogenic differentiation induced by bone marrow mesenchymal stem cells for the treatment of SONFH.

Based on the keywords co-occurrence network, the log-likelihood ratio (LLR) method in CiteSpace was used for cluster analysis of closely related keywords in literature, and related terms were extracted for cluster naming. The results were shown in **Figure 6C**. The 555 keywords in our study formed 11 clusters and 2796 links. The cluster modularity Q value was 0.451 (Q > 0.3 indicates that the clustering structure of the keyword network is significant), indicating that the cluster structure was significant and could represent the research direction of each cluster.In this study, the S value (weighted mean silhouette, S > 0.7 indicates that the cluster members have high similarity.) was 0.7218, indicating that network module keywords have good homogeneity and clustering effect. **Table 3** summarized the information for the largest 11 clusters. Cluster #0 was the largest and contained 95 related references, in which studies focused on the treatment of SONFH by inducing bone regeneration with bone marrow mesenchymal stem cells. Cellular therapy based on mesenchymal stem cells could effectively promote bone repair and angiogenesis in the necrosis area of the femoral head (21). Cluster #1 (size =73) focused on the apoptotic mechanism of steroid-induced avascular osteonecrosis in patients with systemic lupus erythematosus treated with glucocorticoids. Based on the mechanism of apoptosis, scholars explored the effects of bone marrow transplantation on the prevention of SONFH (22). The hotspots in Cluster #2 were the genome-wide association studies, which mainly focused on the genetic polymorphism of SONFH patients, mostly in the form of case-control studies. Some pivotal regulatory genes/proteins such as LRG1, SERPINE2, and STMN1 in the pathogenesis of SONFH were detected (23). Cluster #8 had the highest S value of 0.914 among the 11 clusters, investigated the effects of glucocorticoid and the endothelin-1 (ET1) on human osteocyte metabolism (24), and the main method was a follow-up study. The latest cluster was #3 with a mean year of 2013 and a size of 62. The research hotspots were apoptosis and oxidative stress mechanism of SONFH, the result showed that LncRNA EPIC1 could protect human osteoblasts from dexamethasone-induced cell death (25).

**Table 3:** Summary of the largest 11 keywords clusters.

Cluster-ID	Size	Silhouette	Mean (Year)	Label (LLR)	Included keywords (top 5)
0	95	0.6	2010	stem cells	stem cells; mesenchymal stem cell; bone tissue engineering; bone regeneration
1	73	0.669	2006	Steroid induced osteonecrosis	steroid induced osteonecrosis; systemic lupus erythematosus; avascular necrosis; apoptosis; idiopathic osteonecrosis
2	69	0.608	2012	association study	association study; avascular necrosis; susceptibility; polymorphism; case-control study
3	62	0.67	2013	apoptosis	apoptosis; inhibition; oxidative stress; osteoblast; damage
4	61	0.816	2001	blood flow	blood flow; smooth muscle cell; renal transplantation; femoral head; animal models
5	50	0.763	2008	necrosis	necrosis; therapy; osteonecrosis of the femoral head; acute lymphoblastic leukemia; femoral head
6	35	0.839	2005	peroxisome proliferator-activated receptor-gamma	peroxisome proliferator-activated receptor-gamma; messenger RNA; RNA interference; differentiation induced gene; adenovirus vector
7	30	0.844	2009	mechanism	follow up; bone mineral density; bone loss; gsk3 beta; osteoporosis
8	30	0.914	2002	follow up	mechanism; mitochondria; endothelin 1; chemistry; Raman spectroscopies
9	23	0.845	2009	broiler	broiler; knee osteoarthritis; musculoskeletal disorders; endoplasmic reticulum stress; outcome
10	20	0.837	2007	steroid-induced osteonecrosis	steroid-induced osteonecrosis; polylactic-co-glycolic acid; kidney-tonifying yang; receptor tyrosine kinase; transforming growth factor-beta 2

Keyword burst refers to the phenomenon that high-frequency keywords explode rapidly in a certain period. Through the burst of keywords, the dynamic changes of research hotspots over time can be detected, and the frontier fields and development trends of the subject can be analyzed. CiteSpace was used to analyze the bursts of keywords in our study, and the results were shown in **Figure 6D**. A total of 25 burst keywords were detected over the past 30 years from 1992 to 2021. The keyword with the strongest burst strength of 8.57 between 2004 and 2012 was “avascular necrosis”, followed by “osteogenic differentiation” with a burst strength of 7.13 between 2018 and 2021, and “systemic Lupus erythematosus” with the burst strength of 6.79 during 2006 and 2012. The mutation of high-frequency keywords can be roughly divided into two stages by 2012: The first stage was from 1992 to 2012, during which the research hotspots were mainly on pathophysiological mechanisms such as aseptic necrosis, blood flow, avascular necrosis, and fat embolism. The second phase was from 2012 to 2021, research interests in this period mainly focus on genome-wide and cellular molecular studies of oxidative stress, osteogenic differentiation, gene polymorphism, PPAR (peroxisome proliferator-activated receptor) gamma, and apoptosis. From the perspective of the burst process, the keywords of “osteogenic differentiation”, “proliferation”, “PPAR gamma” and “apoptosis” have been keeping the research heat until 2021, becoming the latest research frontier.

## Discussion

We conducted a comprehensive analysis and mining of global SONFH research on the publications, collaboration of the publication countries/regions, institutions, and authors, research hotspots, and development trends by bibliometrics and visualization analysis methods.

### Global research tendency and distribution of publications in SONFH

780 articles and reviews related to SONFH published from 1992 to 2021 were retrieved in this study. We found that the number of publications has increased from 6 in 1992 to 98 in 2020, 75.77% of which were published in the last decade, which indicates that more and more interest has been paid to SONFH-related topics. An important reason for this growth trend was the widespread use of glucocorticoids in various diseases such as immune diseases (26), rheumatoid diseases, psoriasis (27), and COVID-19 (28). The incidence of SONFH was positively correlated with the dose and duration of glucocorticoid use. Moreover, the increase in publications was inseparable from the rapid development of basic medical sciences and clinical trials in recent years, such as molecular biology (6) and genomics research (29). Finally, the etiology of human diseases was becoming more and more complex. The original SARS (30), the current COVID-19 (31), etc. have complex etiologies, which seriously threaten people’s life and health. To save patients on the edge of life, it was inevitable to use corticosteroids of one kind or another. Therefore, it also buried a lot of hidden trouble for SONFH (32). Notably, the number of global publications in 2021 did not increase compared with that in 2020, which may be related to the global outbreak of COVID-19 in 2020. Corticosteroids have a good inhibitory effect on inflammatory factors and are often used as an adjuvant treatment for viral pneumonia. They have been widely used in the treatment of SARS and MERS (Middle East respiratory syndrome). Corticosteroid treatment is a double-edged sword, and its use in viral pneumonia has been controversial in academia. After the outbreak of COVID-19, the use of corticosteroids has once again attracted extensive attention from scholars. Authoritative experts do not recommend the use of glucocorticoids in patients with COVID-19, and short courses of corticosteroids at low-to-moderate dose, used prudently, for critically ill patients (33). Over time, the virulence of COVID-19 has weakened through mutation, there were fewer and fewer severe patients, and the research heat of corticosteroids in COVID-19 had declined.

The global distribution of publications was unbalanced, mainly concentrated in East Asia and North America. China was the largest contributor to SONFH, accounting for 63.99%, followed by Japan (12.56%) and the United States (12.31%). The results of the correlation analysis indicated that the imbalance in the global distribution of publications can be partly interpreted by demographic or economic factors (19). China was a highly productive country on SONFH, especially in the last decade, the number of publications in China had shown a rapid growth trend. One important reason for this growth was considered to be related to large doses of steroid pulse therapy in SARS patients in 2003 (30). Corticosteroid-related adverse reactions occurred in most SARS survivors such as osteoporosis and femoral head necrosis (34), which attracted extensive attention from scholars to SONFH. To further investigate the pathogenesis and risk factors of SONFH, Chinese scholars conducted several long-term follow-up studies of SARS survivors (34–37) and therapeutic drug studies (38, 39).

### Analysis of the research hotspots and frontiers

Cluster and burst analysis of keywords can reveal the research situation, research hotspots of different directions, and the evolution of frontiers in this field (20). The results of this study demonstrated that in the first 10 years, researchers focused on the pathological features and risk factors of SONFH, such as “blood flow”, “ischemic necrosis”, and “nontraumatic osteonecrosis”, but the exact pathogenic mechanism of ONFH remained unknown. At this stage, the study population was mainly focused on renal transplant patients on corticosteroids. SONFH severely affected the patient’s quality of life and more than half of patients required artificial joint replacement (40), and that explained why orthopedics was the main research category during this period. Since then, researchers conducted in-depth research on the pathological mechanism of SONFH, the keywords of “apoptosis”, “oxidative stress”, “osteogenic differentiation”, “expression”, and “mesenchymal stem cells” were the research hotspots of SONFH. Several hypotheses of the pathological mechanism were proposed during this period, and impaired angiogenesis, abnormal apoptosis of bone and osteoblasts (10, 11), thrombosis and fat embolism (13) caused by bone endothelial dysfunction were considered to be the pathogenesis of SONFH. SONFH-related “medicine research experimental” research areas were becoming popular. The correlational study confirmed that the imbalance of osteogenic/adipogenic differentiation of bone marrow mesenchymal stem cells (BMSCs) was closely related to SONFH (41). In the last 10 years, SONFH research has focused on genomic and cellular molecular studies of osteogenic differentiation, gene polymorphism, PPAR gamma, genome-wide, and apoptosis. Relevant studies have revealed that C/EBPα regulates the expression of PPARγ through histone acetylation as an epigenetic mechanism for SONFH (42). Among the various proposed pathogenesis, the mechanism related to vascular endothelial cell damage had gradually become the most convincing hypothesis (6). Bone tissue engineering therapy, represented by co-transplantation of bone endothelial cells and bone marrow mesenchymal stem cells (BMSCs) (21), has become the research hotspot. At this stage, several studies (43–45) have explored biomarkers for early diagnosis of SONFH. Genomics and cell molecular biology of SONFH are the frontiers, which have great potential to become research hotspots in the near future. The pathogenic mechanisms at the molecular level and genetic mechanisms based on gene polymorphism are expected to provide targets for the treatment and prevention of SONFH.

### Limitation

There are some limitations to this study. First of all, in consideration of only English literature in the study, non-English language literature will inevitably be omitted, resulting in analysis bias. Second, because VOSviewer and Citespace cannot analyze the complete text of the publication, certain information may be ignored. Finally, the latest publications in 2022 were not included because they lacked enough time to accumulate a large number of citations, which may influence our conclusions to some extent due to the rapid updating of research hotspots and frontiers.

### Conclusion

The number of publications in SONFH has increased significantly from 1992 to 2021, especially in the last decade. The global distribution of publications was unbalanced, mainly concentrated in East Asia and North America. The research area of “medicine research experimental” has attracted the most attention in the past decade. According to keywords analysis, mechanism study including “osteogenic differentiation”, “proliferation”, “PPAR gamma” “apoptosis”, “oxidative stress”, and “mesenchymal stem cells” were the research hotspots. Genomics and cell molecular biology of SONFH are the frontiers, which are expected to become the research hotspots in the near future.

## Data availability statement

The original contributions presented in the study are included in the article/**Supplementary Material**. Further inquiries can be directed to the corresponding authors.

## Author contributions

CL and HQ designed the study and collected the data. HX assisted with data analysis and interpretation. YH helped with the statistical method. ZY helped with the graphic generation. WY and PX reviewed and corrected the manuscript. All authors contributed to the article and approved the submitted version.

## Funding

This study was supported by Chinese and Western medicine cooperation Project of Shaanxi Provincial Administration of Traditional Chinese Medicine (Number: 2020-ZXY-010).

## Conflict of interest

The authors declare that the research was conducted in the absence of any commercial or financial relationships that could be construed as a potential conflict of interest.

## Publisher’s note

All claims expressed in this article are solely those of the authors and do not necessarily represent those of their affiliated organizations, or those of the publisher, the editors and the reviewers. Any product that may be evaluated in this article, or claim that may be made by its manufacturer, is not guaranteed or endorsed by the publisher.
