# Supplementary material for: Global research trends of steroid-induced osteonecrosis of the femoral head: A 30-year bibliometric analysis
Source: Front Endocrinol (Lausanne). 2022 Oct 17;13:1027603. doi: 10.3389/fendo.2022.1027603 (PMC9618610; doi:10.3389/fendo.2022.1027603)
Supplement: Supplementary file 2 [file Table_2.docx]

Supplementary material 2：

Table 1 Top 5 productive countries/regions, institutions, and authors on SONFH from 1992 to 2021

| Scale | Rank | Names | Records | Proportion (%) | Centrality | h-index | Total citations | Average citations |
| --- | --- | --- | --- | --- | --- | --- | --- | --- |
| Country/Region | 1 | China | 494 | 63.33 | 0.05 | 59 | 16820 | 21.56 |
|  | 2 | Japan | 97 | 12.44 | 0.00 | 33 | 2946 | 30.37 |
|  | 3 | USA | 95 | 12.18 | 0.20 | 34 | 4287 | 45.13 |
|  | 4 | Germany | 21 | 2.69 | 0.24 | 13 | 466 | 22.19 |
|  | 5 | Canada | 14 | 1.80 | 0.05 | 9 | 455 | 31.79 |
| Institution | 1 | Shanghai Jiao Tong University | 53 | 6.80 | 0.02 | 18 | 998 | 18.83 |
|  | 2 | Chinese Academy of Medical Sciences Peking Union Medical College | 34 | 4.36 | 0.04 | 17 | 611 | 17.97 |
|  | 3 | Xi’an Jiao Tong University | 34 | 4.36 | 0.05 | 13 | 421 | 12.38 |
|  | 4 | Huazhong University of Science Technology | 32 | 4.10 | 0.03 | 14 | 575 | 17.97 |
|  | 5 | Peking Union Medical College | 31 | 3.97 | 0.04 | 17 | 532 | 17.16 |
| Author | 1 | Zhang CQ | 25 | 3.21 | 0.06 | 12 | 693 | 27.72 |
|  | 2 | Gao YS | 19 | 2.44 | 0.02 | 8 | 241 | 12.68 |
|  | 3 | Yamamoto T | 17 | 2.18 | 0.00 | 11 | 693 | 40.76 |
|  | 4 | Li J | 16 | 2.05 | 0.02 | 10 | 422 | 26.38 |
|  | 5 | Wang KZ | 16 | 2.05 | 0.02 | 12 | 307 | 19.19 |
